# Supplementary material for: Hidden phase in a two-dimensional Sn layer stabilized by modulation hole doping
Source: Nat Commun. 2017 Mar 7;8:14721. doi: 10.1038/ncomms14721 (PMC5343494; doi:10.1038/ncomms14721)
Supplement: Supplementary Information — Supplementary Figures, Supplementary Notes and Supplementary References [file ncomms14721-s1.pdf]

Supplementary Information

Supplementary Note 1: Modulation doping

In the current context, modulation doping implies the existence of ionized dopant atoms (i.e., B, As) below the surface and the accumulation of the corresponding (opposite) valence charges in the surface states. By changing the carrier concentration and doping type in the silicon substrate, the Fermi level position in the substrate can be tuned from the bottom of the conduction band (n-type) to the top of the valence band (p-type). The surface has a smaller band gap and different charge neutrality level. Because the bulk and surface are in thermodynamic equilibrium, i.e., the chemical potential is uniform, any change in the bulk chemical potential will lead to a readjustment of the chemical potential in the surface layer. Such readjustment always implies charge transfer between the surface and the bulk. For the n-type material, excess negative charges are trapped in the surface states and the corresponding electric field gives rise to band bending (Supplementary Figure 1a). For the p-type material, band bending is either very small or absent. Even under perfect flat band conditions, the surface layer has an excess hole density due to the fact that it is in equilibrium with the hole reservoir in the bulk (Supplementary Figure 1b). The presence of excess charges in the surface state bands can be inferred from band alignment measurements as discussed below.

To determine the band alignment near the n-2√3Sn surface, and establish the presence of additional surface state charges, Si 2*p* binding energies were determined from XPS core level spectra recorded at 150 K (the lowest temperature achievable in our XPS system), before and after the growth of the 2√3Sn reconstruction. For the clean Si(111)-(7×7) surface, the distance between the Fermi level and bulk valence band maximum (VBM) *at the surface* is 0.63 ± 0.05 eV (*1*). The measured Si 2*p* core level binding energy of the n-type (7×7) surface is 99.3 eV. After forming of the 2√3Sn reconstruction, the Si 2*p* core level has shifted 0.28 ± 0.05 eV towards lower binding energy, due to the re-alignment of the Fermi level at the surface. Here the error margin corresponds to the precision of the XPS spectrometer. Hence, the Fermi level location *at the surface* is $0.63 - 0.28 = 0.35 \pm 0.07$ eV above the Si VBM or 0.80 ± 0.07 eV

below the Si conduction band minimum (CBM; the Si band gap is 1.15 eV at 150 K). The Fermi level in the n-type bulk is located very close to the CBM and, consequently, the conduction band and valence band bend upward near the surface; see Supplementary Figure 1. This implies the existence of a charge depletion layer below the surface and the presence of n-type carriers that are trapped in the surface states.

To determine the precise value of the surface state band gap as well as the precise locations of the surface state band edges relative to the bulk band edges, we fitted STS $I(V)$ spectra measured at 77 K following the procedures in Ref. 2, see Supplementary Figure 2. We find that the surface state band gap for both n-type and p-type interfaces is 0.45±0.03 eV (77 K). The error margin was determined by fitting several spectra, recorded with different set points and different tips. For the n-type system, the Fermi level is located 0.027 eV above the bottom of the empty surface state band, and the bottom of the empty surface state band is located at 0.80 + 0.03 = 0.83 ± 0.07 eV below the Si CBM. This means that the highest occupied surface state band is located 0.83 + 0.45 = 1.28 ± 0.08 eV below the bulk CBM (or 0.13 eV ± 0.08 eV *below* the bulk VBM). Within the accuracy of our measurements, we can state that the highest occupied surface state is located at or slightly below the bulk VBM. For the p-type system, the Fermi level is located 0.12 eV above the highest occupied surface state band, as listed in Supplementary Figure 2. Applying the alignment between the surface states and VBM obtained on the n-type sample, this indicates a nearly flat band condition for the p-type Si substrates (as sketched in Supplementary Fig. 1b and labelled in Figure 4a), because the Fermi level is located close to the VBM both deep inside the bulk and at the surface. This is consistent with the fact that the Sn-induced Si 2*p* core level shift for the p-type systems on the (√3×√3)-B surface is 0 ± 50 meV. (The Fermi level at the (√3×√3)-B surface coincides with the VBM due to the formation of an acceptor-derived impurity band for this very high concentration of B dopants (*3*)). Furthermore, STM images acquired at -0.1 eV reveal the characteristic double dimer features of the B-2√3Sn phase, meaning that the states involved in the tunneling at this bias are genuine surface states. Because the Fermi levels of the B-2√3Sn and p-2√3Sn systems are located near the bottom of the surface state gap, they contain an excess concentration of holes. Supplementary Figure 1 summarizes our findings.

In the abrupt depletion layer approximation, one can establish a simple relation between the excess charges in the surface states, the band bending and the bulk doping levels. There is no experimental evidence for significant arsenic segregation for n-type Si substrates. In this case, we can approximate the surface doping level from the abrupt depletion layer approximation (*4*), using the nominal doping level of the bulk wafer and the band bending parameters obtained from XPS. Accordingly, we obtain an electron doping level of the order of 1% for the n-type sample surface at 150 K. In the p-type case, however, the boron concentration in the Si substrate is not uniform as boron atoms segregate towards the surface, meaning that such estimates could be far off. In order to obtain more accurate estimates of the various doping levels for the n- and p-type samples, it will be necessary to determine the dopant profiles in the subsurface regions. This is beyond the scope of the current investigation. Nonetheless, qualitatively, we can state that in thermodynamic equilibrium, increased doping levels in the bulk imply increased doping levels in the surface layer.

Supplementary Note 2: The B-2√3Sn double layer structure.

Supplementary Figure 3a shows an STM image and line profile of adjacent B-2√3Sn and Sn/Si(111)(√3×√3)*R*30°-B (B-√3Sn for short) terraces. The former reconstruction has a coverage of 14/12 ML (see main text), while the latter has a coverage of 1/3 ML. In the lower left corner of the image, one can see the original Si(111)(√3×√3)*R*30°-B substrate (dark area), decorated with relatively bright Sn adatoms and Sn adatom clusters. Next to this diluted adatom region, we find a narrow (√3×√3)*R*30° ordered Sn adatom phase (i.e., B-√3Sn). The Sn adatoms in this region appear to be ~1 Å higher than the Si adatoms of the original Si(111)(√3×√3) *R*30°-B surface (*5*). Based on the T_4 registry and apparent height of these Sn atoms, we conclude that they have substituted for the Si adatoms of the Si(111)(√3×√3)*R*30°-B substrate.

The large bright terrace on the right is the B-2√3Sn phase. The B-2√3Sn reconstruction has a significantly larger apparent height relative to the B-√3Sn phase, consistent with the structure model in Figure 1b with the outward relaxed Sn tetramers sitting on top of the first layer. This is

fully consistent with previous reports on 2√3Sn for n-type systems (*5-8*), but inconsistent with the most recent report (*9*). The B-2√3Sn height relative to the B-√3Sn phase was measured for different bias voltages, as shown in Supplementary Figure 3b. The B-2√3Sn phase is about 2.4 Å higher than B-√3Sn for most biases, but the apparent height difference is significantly smaller at small biases, -0.1 V < V_s < +0.5 V. The latter coincides with the energy gap of the B-2√3Sn surface, as resolved by STS in Fig. 4 in the main text. The semiconducting gap of the B-2√3Sn structure reduces the tunneling probability for the corresponding bias range, moving the tip closer to the surface. This gives rise to the lower apparent height in the STM image.

Supplementary Note 3: LEED investigation of the B-2√3Sn melting transition

The n-2√3Sn surface reconstruction melts at about 190 °C and the (2√3×2√3)*R*30° LEED pattern transforms into a (1×1) pattern (*6*, *7, 10*). Because the liquid surface layer produces no diffraction spots, only the integer order beams from the substrate remain visible. In Supplementary Figure 4, we track the melting transition of the B-2√3Sn structure by recording the intensity of the 2√3 spots as a function of temperature, using a CCD camera. Here, the Sn layer melts at about 184 °C. The melting transition exhibits a 12 °C temperature hysteresis, indicative of a first order transition (as expected). Hence, the melting temperature of the 2√3Sn phase on the n-type Si(111) and p-type Si(111)(√3×√3)*R*30°-B substrates are identical within the margin of error.

The (√3×√3)*R*30° LEED pattern persists well above the melting temperature, which indicates that the subsurface boron atoms remain located at the S_5 lattice sites as the Sn layer melts. The identical melting temperatures strongly suggest that the structure of the 2√3Sn reconstruction is identical for the n-type and p-type substrates. The boron atoms remain firmly in place, even when the Sn layer melts, meaning that they do not chemically mix with the Sn layer. This is consistent with the identical STM images of the 2√3 structures on n-type and p-type Si in Fig. 2 of the main text.

Supplementary Note 4: LEED *I*(*V*) comparison of the B-2√3Sn and n-2√3Sn structures

LEED data were collected using an Omicron SpectaLEED system. EasyLEED software (*11, 12*) was used to extract the beam intensities as a function of beam energy. Intensities were symmetry averaged. Supplementary Figure 5 presents the beam energy dependent spot intensity of the (0,1) and (0,3/2) beams. The position of the maxima in these LEED *I*(*V*) profiles is nearly identical, supporting our conclusion that the structure of the 2√3Sn reconstruction is not affected by the presence of a subsurface (√3×√3)*R*30°-B structure, although structural determination from these data are beyond the scope of this investigation.

Supplementary Note 5: STM assisted formation of the B-4√3Sn and p-4√3Sn structure

A B-2√3Sn surface freshly prepared on a (√3×√3)*R*30°-B reconstructed substrate is initially scanned with negative bias. Panel a in Supplementary Figure 6 shows the very first scan of this surface. The B-2√3Sn phase is preserved as long as the surface is scanned at negative bias, regardless of the applied tunneling current. A large fraction of the surface transforms to the B-4√3Sn phase when switching to positive sample bias, even for tunneling currents as small as 20 pA (panel b and the 4th data point in panel g). The area fraction of the B-4√3Sn phase will stay within the range of 60% - 80% in subsequent scans, regardless of the bias polarity or tunneling current (panel g). The system never reverts back to the initial B-2√3Sn structure. This means that the initial conversion of the fresh surface from a pure B-2√3Sn phase to a mixed phase is irreversible at 4.4 K. More specifically, while the B-2√3Sn to B-4√3Sn transition can be reversible locally under the STM tip, the transition from the pure B-2√3Sn phase to the mixed phase is irreversible on a larger scale. Very similar tip-assisted 4√3Sn formation behavior is observed for the p-2√3Sn surface at 5 K: the fresh surface is fully covered by p-2√3Sn phase if scanned at negative bias (panel (e)). Upon switching to positive bias (panel f), most the surface will transform to the 4√3Sn phase. In contrast, the electron doped Sn surface always show a pure 2√3Sn phase, regardless of the scanning parameters

(see STM images at 5 K with negative bias in Fig. 2c and with positive bias in Supplementary Fig. 7).

After the initial conversion to the mixed phase, the area fractions of the coexisting B-2√3Sn and B-4√3Sn phases are slightly dependent on the tunneling bias during subsequent scans. This is illustrated in panel g. Specifically, the area fraction of the B-4√3Sn increases (decreases) when switching to positive (negative) bias. However, the area fractions still remain within the 60%-80% range for tunneling currents up to 2 nA. Scanning with even larger tunneling current, *e.g.*, larger than 10 nA, could give rise to changes beyond the 60% - 80% range, but the surface and/or tip are easily destroyed under those conditions. Most importantly, after the initial irreversible conversion to the mixed phase at 4.4 K, we never were able to recover a homogenous B-2√3Sn phase (nor a homogeneous B-4√3Sn phase). This behavior was reproduced for the 0.03 Ω•cm p-type 7×7 reconstructed Si substrate (data not shown).

Because the area fraction of the 4√3Sn phase depends on the scanning bias, tunneling current, as well as the scanning history, it is essential to determine the area fractions (or 4√3 order parameter; see Fig. 2f) in a systematic manner in order to establish meaningful comparisons between the different substrates and temperatures. The order parameters of 4√3Sn in Fig. 2f of the main text are determined from STM images scanned in dual bias mode (±1.5 V, 200 pA), meaning that the tunneling polarities are reversed during forward and backward scans for each line of the image while the voltage amplitude and the tunneling current remain the same. The fast switching of the bias polarity in dual bias scanning effectively averages the bias polarity effect seen in sequential imaging (Supplementary Figure 6). The domain configurations are found to be identical in forward and backward scans even with opposite bias polarities, presumably due to the slow kinetics of the small reversible part of the phase change. (The latter is evident from the slow variation of the area fraction between the 7th – 9th (or the 10th -12th) data points in panel g). The area fraction determined from the dual bias images roughly equals the average value of the area fractions obtained from the corresponding two single-bias images (positive and negative bias images acquired sequentially, such as Supplementary Figures 6c and 6d), indicating that the bias polarity effect

is minimized in the dual bias scheme. The actual area fractions in Fig. 2f were determined after repeated dual bias scans until the area fraction was fully stabilized.

The mechanism of the tip assisted domain conversion is not clear. Generally, one considers two possible scenarios. One mechanism involves transient doping due to charge injection from the STM tip (*13*, *14*), which could play a role when the injected charges don't tunnel or diffuse fast enough into the bulk. This can happen at low temperature where the bulk conductivity is low. The other mechanism involves the electric field effect (*15*). In both cases, one would have to consider how these mechanisms alter the total energy of the B-2√3Sn and B-4√3Sn phases, as well as that of the unknown intermediate structure or 'transition state'. The difference between these energy levels determines the height of the activation barrier and resulting probability of the structural conversion (*16*).

The 4√3Sn phase is clearly stabilized by hole doping with respect to the 2√3Sn phase. Yet, the tip assisted conversion from 2√3Sn to 4√3Sn on p-type samples only happens in empty-state imaging, suggesting that it may require injection of negative charge carriers. To achieve such conversion, the energy difference between the 2√3Sn phase and the transition state would need to be reduced by electron injection. One possibility is that the total energy of the 2√3Sn phase increases relative to that of the transition state with electron doping, which seems plausible considering the fact that these electrons occupy states above the band gap. Alternatively, an electric field effect or electron doping could reduce the total energy of the transition state.

The electric field effect likely plays a significant role. Here we note that the 2√3Sn has a polar symmetry (see Fig. 3), meaning that it could support a macroscopic electric polarization that would couple to an external electric field. In such a case, one would classify the transition as a ferroic transition. Note, however, that the 'polarity' of the 2√3Sn is not switchable due to the fact that the (111) substrate lacks two-fold rotational symmetry.

We finally note that STM induced phase transformations have been seen in other systems (*13-18*). However, all of them turned out to be reversible, even below 10 K (*13, 14, 17*),

whereas here, the conversion is irreversible and the B-4√3Sn configuration is static. Moreover, none of these systems involve a transition between polar and non-polar symmetries.

Supplementary Note 6: Intermediate doping levels

In addition to the 2√3Sn double layer reconstruction grown on the n-type and the (√3×√3)*R*30°-B substrates, we also prepared the 2√3Sn reconstruction on three different p-type Si substrates, two of them exhibiting a boron deficient (√3×√3)*R*30° reconstruction (0.004 and 0.009 Ohm•cm), and one of them exhibiting a (7×7) reconstruction (0.03 Ohm•cm); see the Materials and Methods section. Since the chemical potential of these surfaces is still located near the bulk valence band maximum, the p-2√3Sn reconstruction is still expected to be hole-doped (Supplementary Note 1). As seen in Supplementary Figure 8, all of these surfaces condense into the mixed phase morphology.

It is difficult to estimate the precise amount of charge doping in the Sn layer on these p-type substrates, since B dopants segregate to surface when heating and flashing these substrates in UHV. Boron segregation will lead to higher (unknown) hole concentrations just below the surface, up to a maximum of one hole per (√3×√3)*R*30°-B unit cell. This, in turn, translates into about 0.29 holes per Sn atom but we emphasize that without detailed electronic structure calculations, it is not certain in which orbital or band the holes reside.

Supplementary Note 7: Dopant inhomogeneities from conductance mapping.

In Supplementary Notes 3 and 4, and in the main text we have shown that there is no structural modification of the B-2√3 structure due to boron incorporation. This exclusion was based on the nearly identical LEED *I*(*V*) spectra for the 2√3 phases on the n- and (√3×√3)-B substrates, their nearly identical melting temperature, and their exactly-identical appearance in STM images. The only physical difference appears to be the location of the chemical potential (Figure 4a). Of course, it is possible that the structure modifications are too small to

be detected by the above techniques. However, such a structural modification would by far be the largest for the (√3×√3)-B substrate with the 1/3 ML boron underlayer, and should be significantly smaller for the 2√3Sn film grown on the p-type Si(111)7×7 (p-2√3Sn) which has no boron at the S_5 lattice location. In this case, it would be very difficult to rationalize why the 4√3 domains are striped with nearly identical width distribution for the (√3×√3)-B and 7×7 substrates (see Supplementary Note 6). Instead, we argue that the hole-doped surface phase is inherently phase separated into striped domains where the stability of the striped phase is determined by the doping level.

One may question whether the formation of the mixed phase correlates with a possible ordering of the boron dopants, since the dopant atoms underneath the Sn layer are not directly visible in STM images. In particular, could the formation of the 4√3 domains be triggered by a possible stripe formation of boron below the surface? Such patches would be very heavily hole-doped while regions in between these boron patches are relatively depleted of holes. This should give rise to lateral variations in the tunneling conductance at a bias that is sensitive to subsurface states. To verify or refute this possibility, we collected d*I*/d*V* maps (or conductance maps) of the 2√3 surface at different doping levels at 77 K, *i.e.*, just above the transition temperature to the 4√3 structure. Supplementary Fig. 9 (right column) compares the d*I*/d*V* maps of 2√3 surfaces grown on the n-type and the three p-type Si(111) substrates with different boron concentrations. The tunneling bias is -0.4 eV for the p-type and -0.75 eV for the n-type samples, which corresponds to the location of the highest occupied surface state peak in the d*I*/d*V* spectra in Fig. 4a. This state is degenerate with the bulk valence band continuum and, consequently, we should be more sensitive to the subsurface region. None of the d*I*/d*V* maps (panels b, d, e, f) show any evidence of striped-like patterns in the tunneling conductance, and hence a stripe-like aggregation of subsurface boron is highly unlikely. There is some disorder/inhomogeneity in the d*I*/d*V* maps of the 2√3 surface, grown on the boron deficient and boron saturated (√3×√3)-B substrates. This can be attributed to some inhomogeneity in the distribution of subsurface boron atoms (*19*).

The observation that the domain width distribution and saturation fraction of the 4√3Sn structure do not depend on the density of boron atoms directly below the surface, as discussed

in Supplementary Note 6 and Supplementary Figure 10, and Fig. 2f, and the fact that the dI/dV maps show no pattern formation at 77 K strongly indicates that the emergence of the mixed phase does not depend on the actual placement of the subsurface boron atoms, but must instead be triggered by the change in the chemical potential (i.e., hole doping).

Supplementary Note 8: Domain width distribution

The coexisting 2√3Sn and 4√3Sn domains are very anisotropic in shape. They are elongated along the $<\bar{1}\bar{1}2>$ direction and quite narrow along the perpendicular $<\bar{1}10>$ direction, as seen in Fig. 2d and Supplementary Figure 6. The histograms in Supplementary Figure 10 present the distribution of domain widths along the $<\bar{1}10>$ direction from STM images recorded at 4.4 K, for the Sn double layers grown on a (√3×√3)R30°-B reconstructed surface (panels a and b) and on a p-type (7×7)-reconstructed substrate (panel c and d). Unit cells are indicated in Fig. 3. 4√3Sn phase domains with half integer width (equivalent to odd multiples of 2√3 unit cells) are occasionally observed and are excluded from the histogram. The domain width distributions are well fitted by an exponential decay ($y = A \cdot \exp\left(-x / x_0\right)$), as shown by the solid lines. Considering the fact that the distribution of intervals, either for time or space, of random events is generally characterized by an exponential distribution, the observed exponential decay is consistent with random domain wall spacing. In contrast, long-range elastic or electrostatic forces are expected to lead to preferred domain widths. It should be noted that the Sn layer grown on the (√3×√3)-B surface has a much larger (up to 1/3 ML) concentration of near surface B dopants than that grown on the lightly doped p-type Si(7×7) surface (minimal segregated B dopants underneath the Sn layer). However, their 4√3Sn domain width distributions are essentially identical within the error margin (panels b and d of Supplementary Note 6): the averaged domain width obtained from the fitting is 2.2 unit cells for p-4√3Sn, and 2.5 unit cells for B-4√3Sn. This is consistent with their identical 4√3Sn saturation coverage at 4.4 K (Fig. 2f). This observation excludes any hypothetical 4√3Sn formation mechanism that directly relies on local structural

modifications due, or accumulation of B dopants underneath the Sn layer, which is discussed more in Supplementary Note 9.

Supplementary Note 9: Breaking of the *p2gg* space group symmetry in the 4√3Sn structure

STM images of the 4√3Sn structure in the main text suggest *p2gg* (rectangular) 2D space group symmetry (*20*), implying the existence of glide plane symmetry along the $<\bar{1}10>$ and $<\bar{1}\bar{1}2>$ directions. However, because the Si(111) substrate lacks two-fold rotational symmetry, the space group must be reduced to *pg*. This implies that the two up atoms of the Sn tetramer in the 4√3Sn structure are no longer equivalent (same for the two down atoms). While this is not seen in the STM image in Fig. 3, the symmetry breaking is evident from the image in Supplementary Figure 11 which is acquired with different tunneling parameters. In this image, showing coexisting B-2√3 and B-4√3 regions, only the up atoms are seen. The bright dimers of the B-2√3Sn structure remain symmetric with respect to the *cm* mirror plane symmetry. On the other hand, the two up atoms of the B-4√3Sn phase are clearly inequivalent, forming non-symmetric dimers that are oriented 45° relative to the dimers of the B-2√3Sn phase. Hence, the 4√3Sn phase does not have two fold rotational symmetry, meaning its space group is *pg*. Note that *pg* (2D space group #4) is a subgroup of *cm* (#5), while *p2gg* would be a higher space group (#8). Only a *cm* to *pg* transition would be consistent with the notion of spontaneous symmetry breaking.

Supplementary Note 10: Electron doping by the adsorption of alkali atoms

Adsorption of alkali atom has been widely used to dope surface structures with excess electrons. As shown in Supplementary Fig. 12, the 4√3Sn phase area fraction at 60 K decreases from ~40% (panel a) to ~ 1 % after the adsorption of ~ 0.005 ML of potassium on

the B-2√3Sn surface (panel b). Moreover, d*I*/d*V*/(*I*/*V*) data from the 2√3Sn phase show almost identical features before and after the adsorption of potassium, except for a ~ 0.2 eV shift toward higher binding energy (panel c) for the latter, consistent with an upward shift of the surface chemical potential due to electron doping. Identical results are obtained by replacing potassium with cesium atom (data not shown). These data confirm that the tip-assisted phase transformation on the B-2√3Sn surface is caused by hole doping; the electrons doped into the surface by the alkali atoms compensate the modulation-doped holes from the substrate, thereby decreasing the relative stability of the 4√3Sn phase.

We note that, besides charge doping, the adsorption of alkali dopant atom on the surface unavoidably introduces chemical or structural disorder (panel b) that could in principle influence the intricate balance of the competing phases. Subsurface modulation doping is therefore preferred over surface decoration with electropositive or electronegative species.

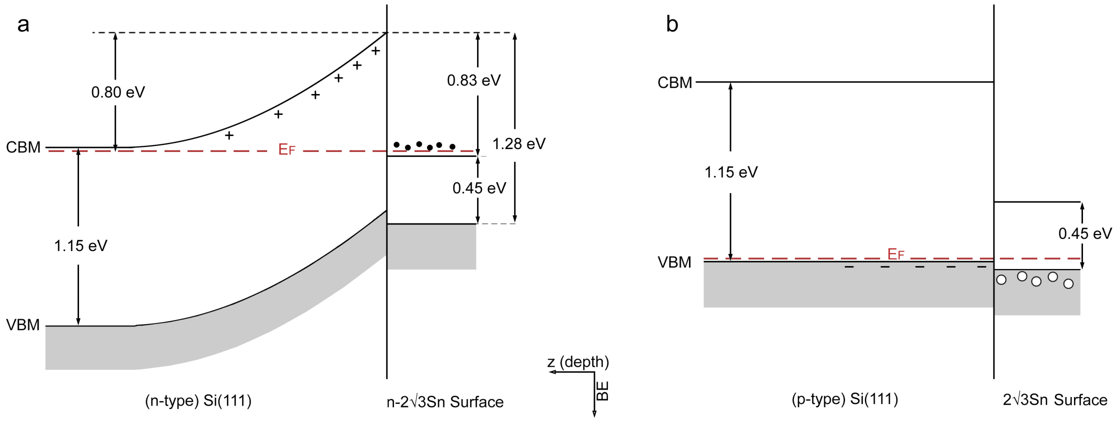

Supplementary Figure 1| Band bending diagram. Band alignment diagram for the n-2√3Sn (**a**) and p-2√3Sn (**b**) surfaces, as determined from XPS measurements of the Si 2*p* core level binding energies and scanning tunneling spectroscopy. Energies are relative to the Fermi energy (red dashed line) at 0 eV. The charges drawn in the silicon in panels (a) and (b) are those of the ionized donors or acceptors, respectively. Opposite valence charges are located in the surface layers.

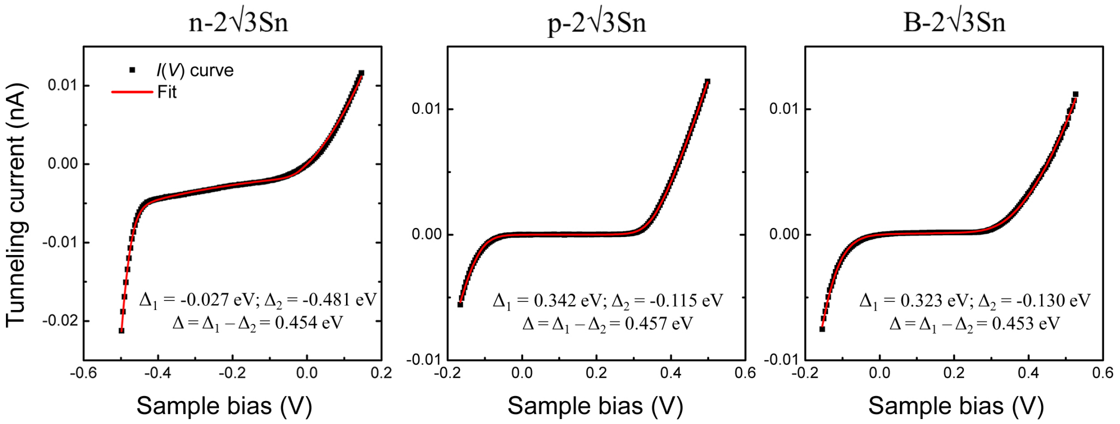

Supplementary Figure 2 | Fitting of the *I*(*V*) spectra of the n-2√3Sn, p-2√3Sn, and B-2√3Sn surfaces associated with their d*I*/d*V*/(*I*/*V*) curves in Fig. 4a. The surface state band gap information could be obtained from fitting the *I*(*V*) curve near the surface band gap regime: $\Delta_{1(2)}$, which is the energy difference between the lowest unoccupied (highest occupied) surface state and the Fermi level, and therefore the total band gap is $\Delta = \Delta_1 - \Delta_2$. The fitted curve (red) are superposed with the *I*(*V*) curve (black dotted), and the parameters obtained from the least square fits are listed in each graph.

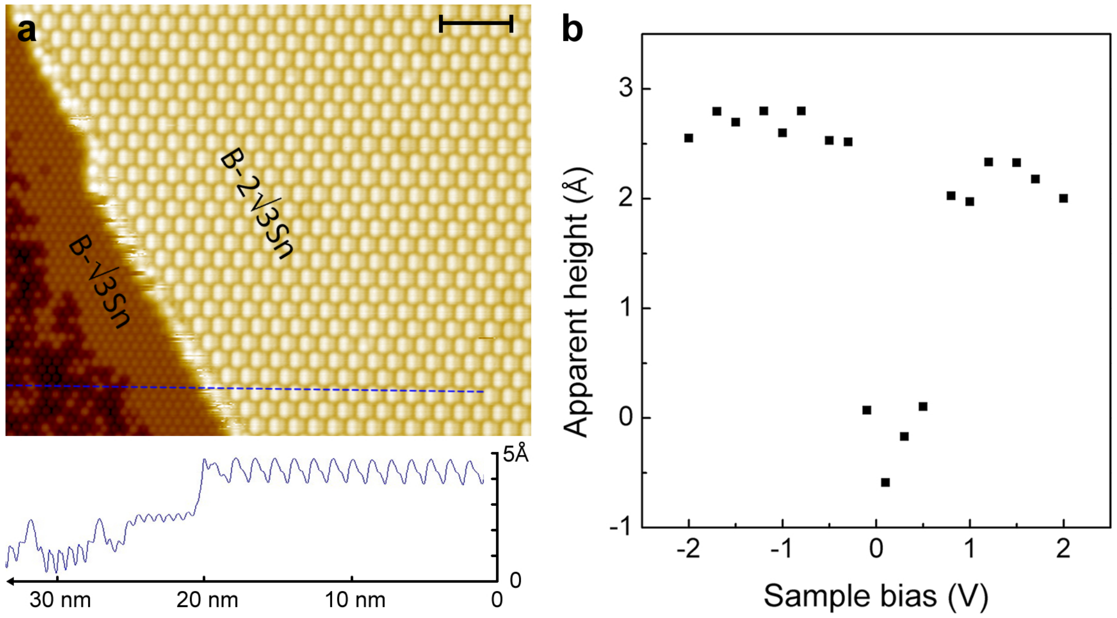

Supplementary Figure 3 | Registry and apparent height of the B-2√3Sn phase. (**a**) STM image and line profile of the various surface structures following the deposition and annealing of ~ 1 ML of Sn on Si(111)(√3×√3) *R*30°-B. The image is scanned at V_s = 2 V, I_t = 0.2 nA, at room temperature. The scale bar is 5 nm. (**b**) Bias dependent apparent height differences between B-2√3Sn and B-√3Sn domains grown on the Si(111)(√3×√3) *R*30°-B substrate. The height differences are extracted from line profiles crossing a domain boundary between the B-2√3Sn and B-√3Sn phases in various STM images. The images are acquired at 77 K with various bias voltages and a constant tunneling current of 0.5 nA, except for the lowest biases which were recorded at a lower tunneling current so as to avoid crashing of the STM tip. The +/- 0.3 V images were taken at 0.1 nA and the +/- 0.1 V images were taken at 0.02 nA.

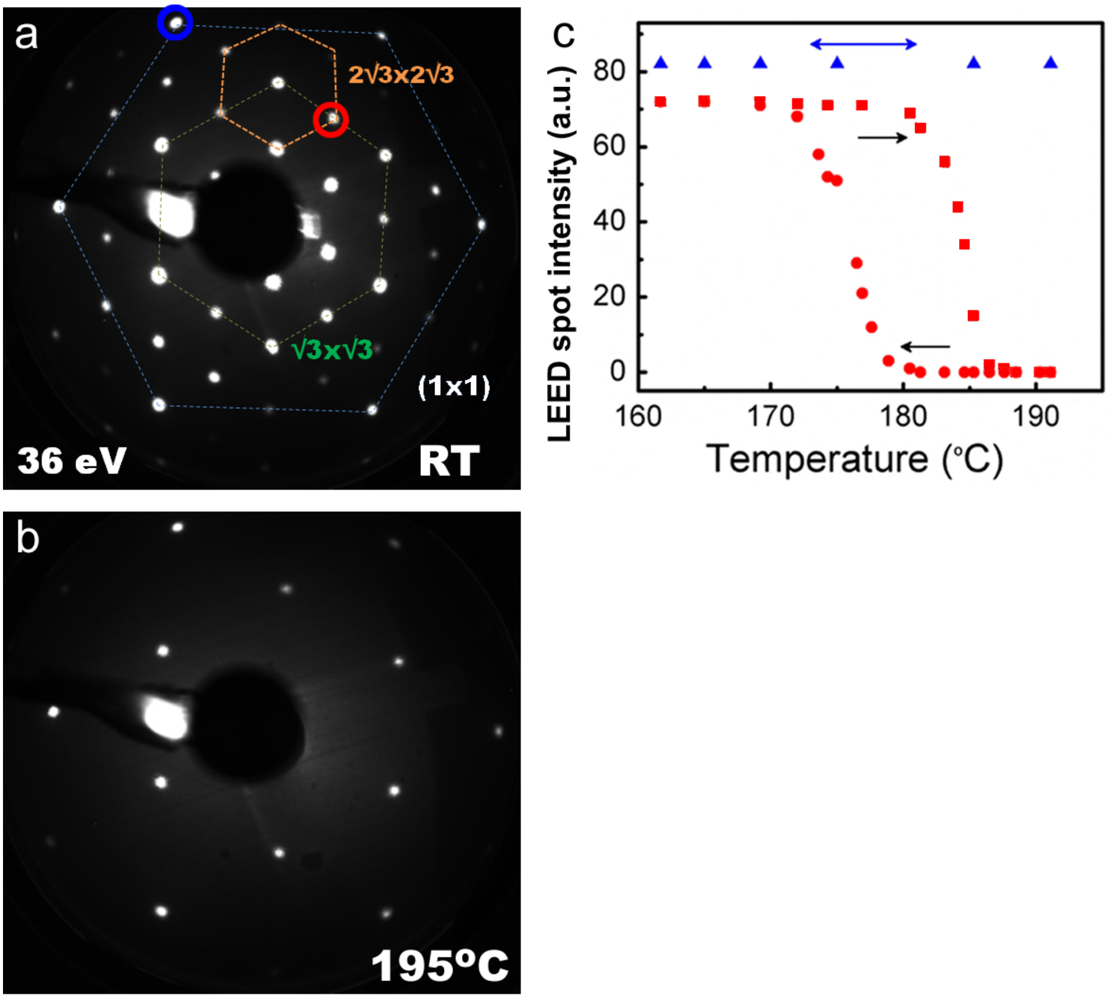

Supplementary Figure 4 | LEED measurements across the melting transition of the B-2√3Sn phase. (**a**) LEED pattern captured at room temperature. One of the (2√3×2√3)R30° and 1×1 reflections are circled in red and blue, respectively. (**b**) LEED pattern taken at 195 °C, *i.e.*, above the melting temperature. Note that the reflections specific to the 2√3 structure in (**a**) have disappeared. Only the 1/3 order beams of the (√3×√3)R30° reconstruction remain present. (**c**) Intensity of the 2√3 beam, circled in panel (a), recorded during heating and cooling (red squares and circles, respectively). Blue triangles correspond to the (1×1) bulk reflection.

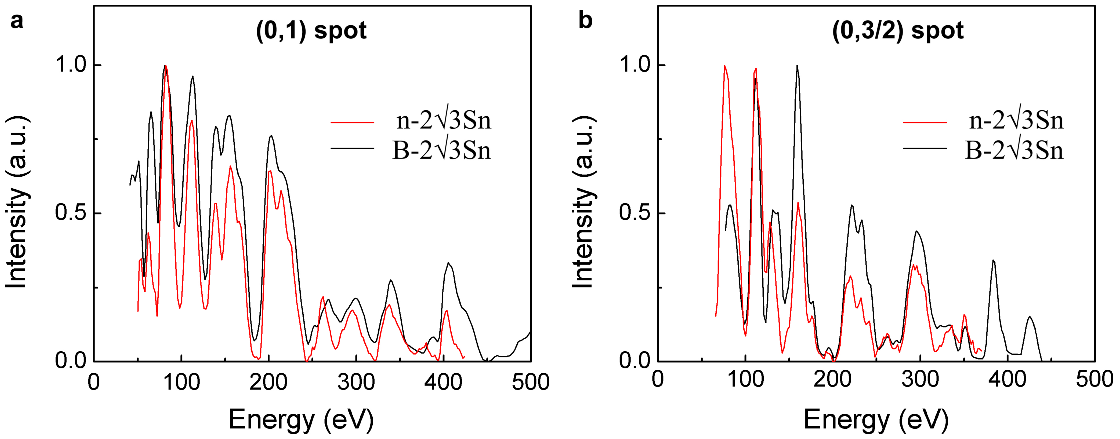

Supplementary Figure 5 | Comparison of LEED *I*(*V*) curves measured on the n-2√3Sn and B-2√3Sn surfaces. LEED *I*(*V*) curves are plotted for the (**a**) (1,0) and (**b**) (0,3/2) diffracted beams. The data were recorded at 150 K for n-2√3Sn and at 40 K for B-2√3Sn, with a beam energy step size of 2 eV.

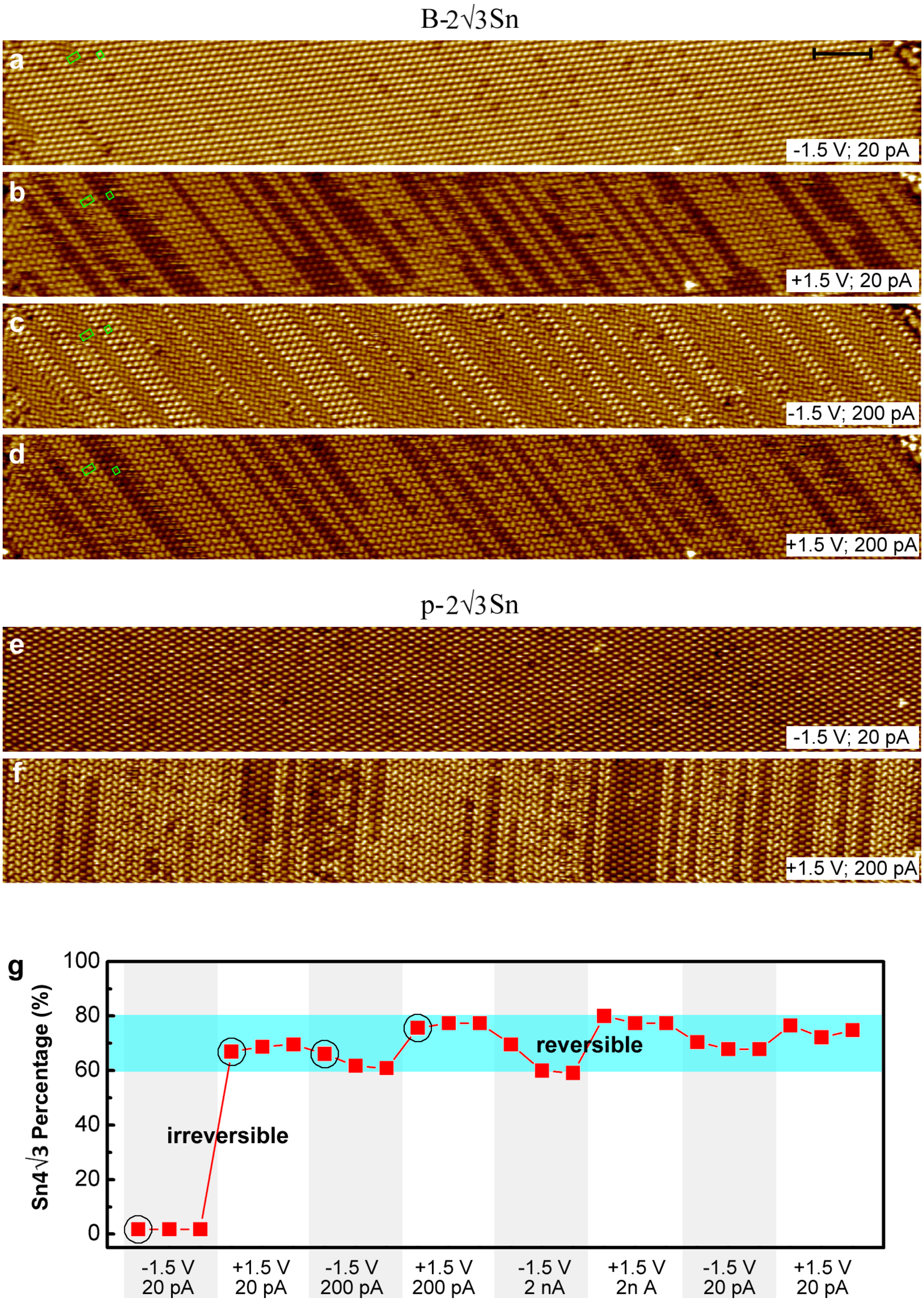

Supplementary Figure 6 | STM tip-assisted phase transformation at 4.4 K. The scale bar in (**a**) applies to all STM image panels and is 10 nm. STM images acquired at 4.4 K and recorded with different tunneling parameters, for a B-2√3Sn sample (**a-d**), and for a p-2√3Sn sample (**e-f**). **(g)** Area fractions of the B-4√3Sn phase, determined from the full series of

images in (**a-d**)**.** Each data point corresponds to an STM image recorded with a single bias. The tunneling parameters are indicated along the horizontal axis. Going from left to right, data points represent sequential images. The first data point represents the very first scan of the surface area. Three images are repeatedly scanned under the same tunneling conditions to make sure that the B-4√3Sn fraction is stabilized. The circles correspond to the images shown in (**a-d**). Reversible and irreversible changes are indicated. The range of reversible changes is highlighted in blue. The area fraction of 4√3Sn phase in (**f**) is ~ 71 %.

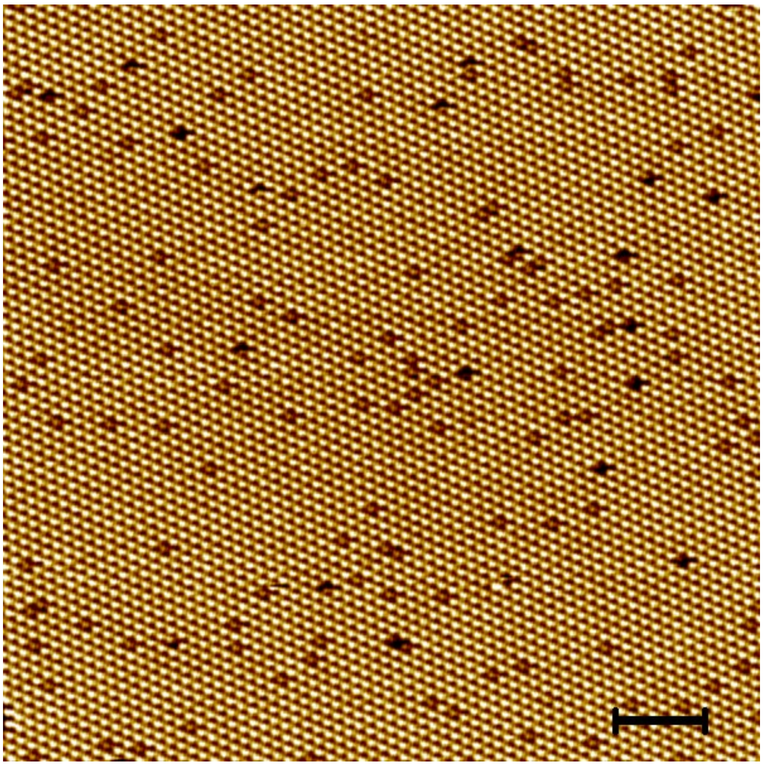

Supplementary Figure 7 | A large STM image of the Sn double layer grown on n-type Si, at 5 K. V_s = 3 V, I_t = 0.005 nA. The scale bar is 10 nm. Despite being recorded at a positive tunneling bias where hole-doped samples exhibit the phase transformation to the mixed phase, n-type samples remain a in the 2√3Sn phase.

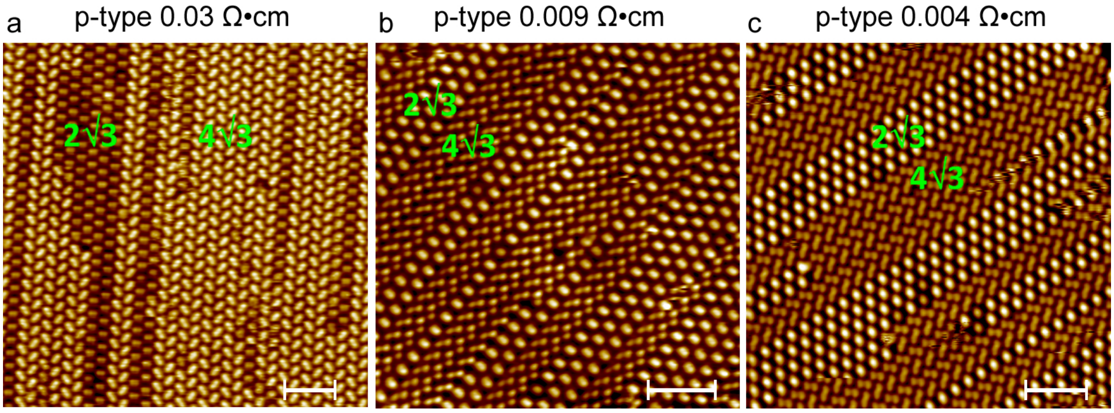

Supplementary Figure 8 | STM images taken at 4.4 K of the 4√3Sn reconstruction for different hole doping. Nominal room temperature resistivities are indicated. All surfaces show a mixture of two phases: Si(111)(2√3×2√3)*R*30°-Sn and Si(111)(4√3×2√3)*R*30°-Sn, identical to the observations when using the Si(111)(√3×√3) *R*30°-B substrate (Fig. 2d). The imaging conditions are (**a**) V_s = 1.5 V, I_t = 0.2 nA; (**b**) V_s = -1.5 V, I_t = 0.2 nA; and (**c**) V_s = -2 V, I_t = 1 nA. All scale bars are 5 nm.

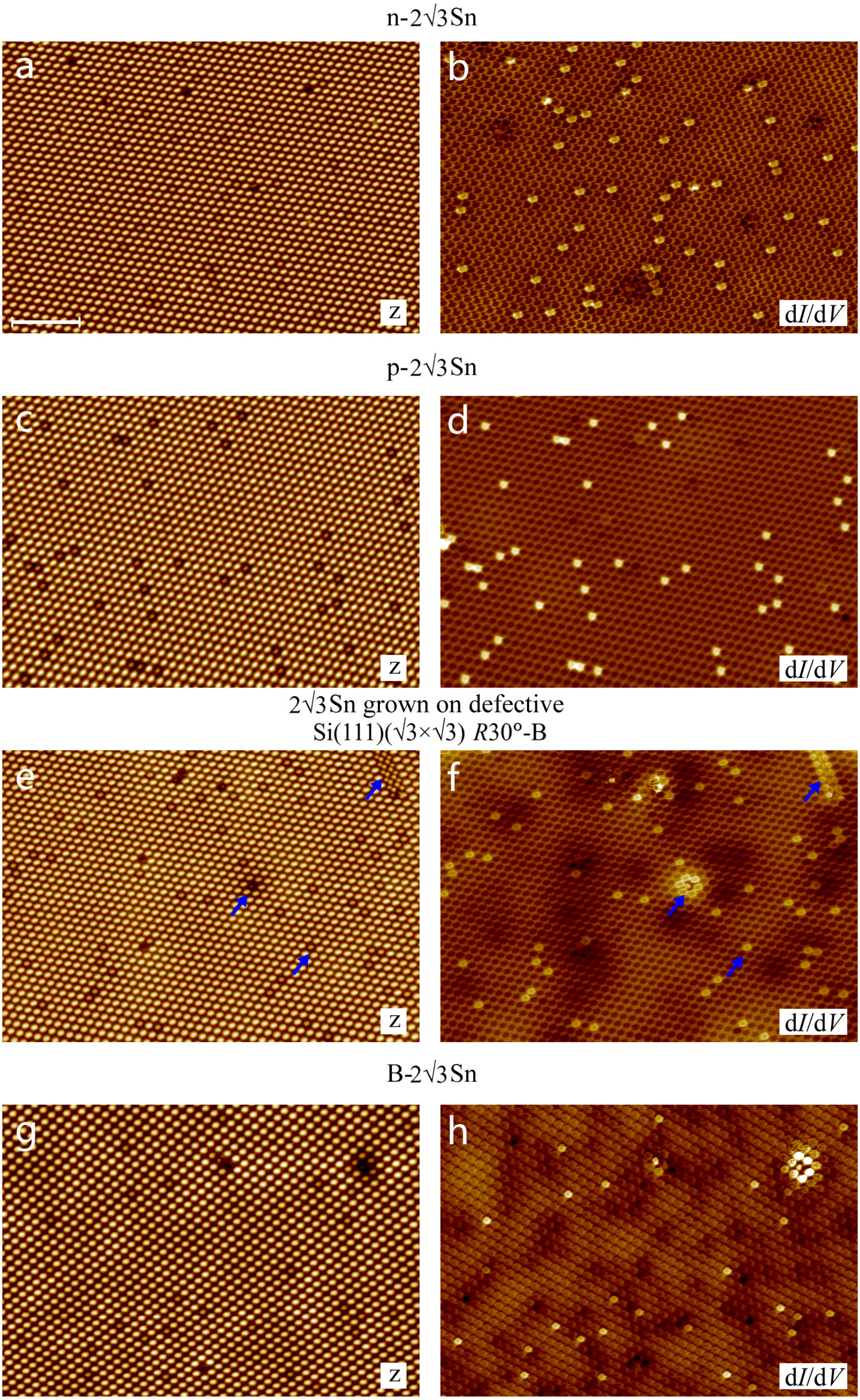
n-2√3Sn
a
b
z
dI/dV
p-2√3Sn
c
d
z
dI/dV
2√3Sn grown on defective
Si(111)(√3×√3) R30°-B
e
f
z
dI/dV
B-2√3Sn
g
h
z
dI/dV

Supplementary Figure 9 | d*I*/d*V* mapping of the 2√3Sn surface for different doping levels. Left column contains topographic STM images taken simultaneously with the d*I*/d*V* maps in the right column. The data were recorded at 77 K, just above the transition temperature to the 4√3 structure. All images are identical in size, and the scale bar in panel (**a**) is 5 nm. The 2√3Sn surface are grown on: (**a**,**b**) n-type Si(111)-(7×7), (**c**,**d**) p-type Si(111)-(7×7), (**e**,**f**) a boron deficient (√3×√3)*R*30°-B reconstructed surface, and (**g**,**h**) a well ordered (√3×√3)*R*30°-B surface with a saturated subsurface boron content (~1/3 ML). The scanning bias is -0.75 V for the n-type substrate (**a**,**b**) or -0.4 V for the p-type substrates (**c-h**), and the tunneling current is 1.0 to 1.5 nA. The arrows in panels (**e**) and (**f**) mark a short 4√3Sn patch (upper arrow) and two common surface defects (lower two arrows). One type of defect (marked with the lowest arrow) is found in all topographic images and shows up as a single bright unit cell in the d*I*/d*V* maps.

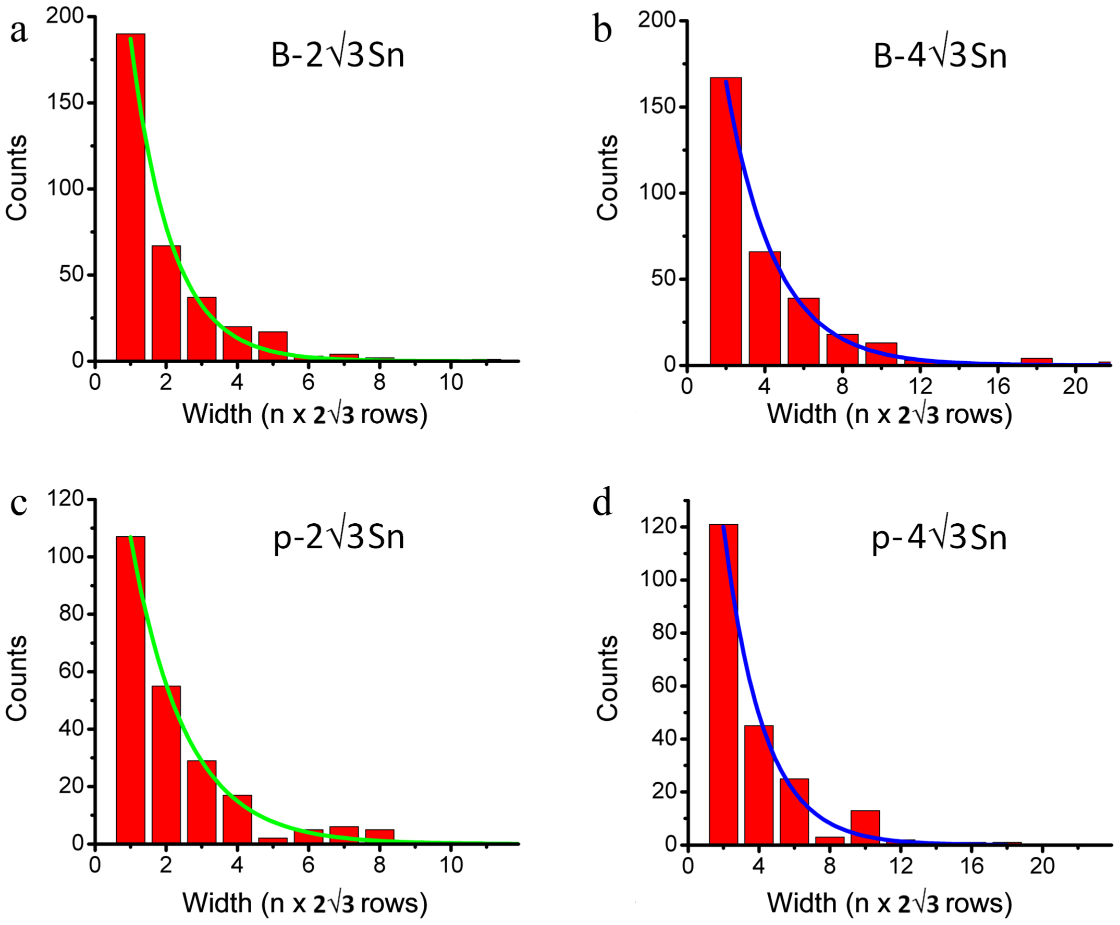

Supplementary Figure 10 | Statistics of the domain widths of the coexisting 2√3Sn and 4√3Sn phases at 4.4 K. Panels (**a**) and (**b**) show the domain width distributions on the (√3×√3)*R*30°-B substrate. Panels (**c**) and (**d**) show the domain width distributions on the p-type Si(111)-(7×7) substrate.

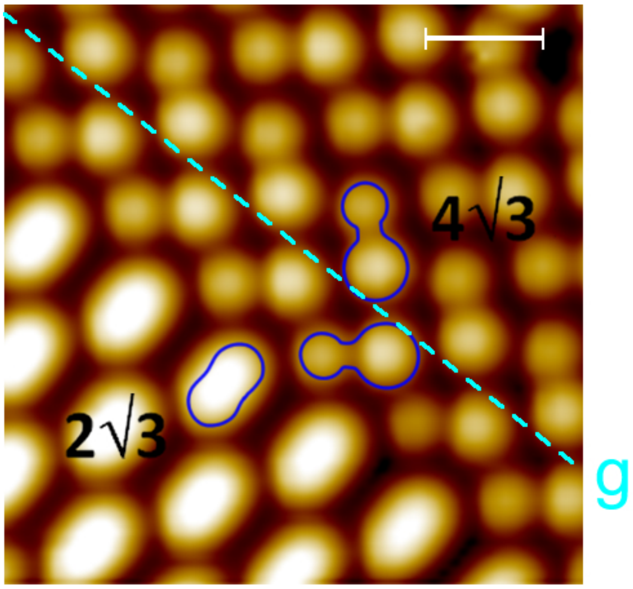

Supplementary Figure 11 | STM image of coexisting B-2√3Sn and B-4√3Sn phases, obtained at 4.4 K. This image was acquired with a bias voltage of -0.5 V and a tunneling current of 0.15 nA. The dashed line marks a glide axis of the B-4√3Sn structure. The scale bar is 1 nm.

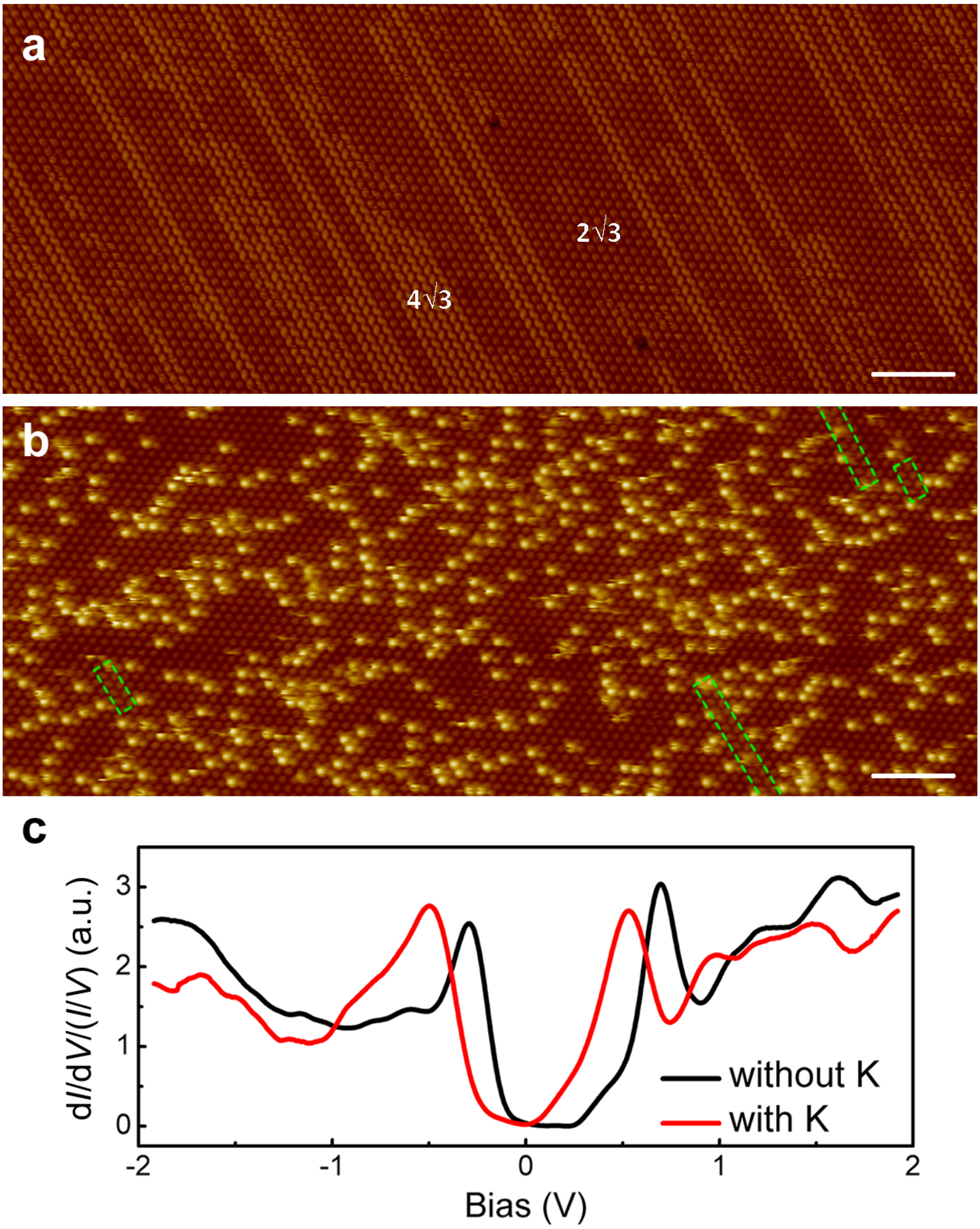

Supplementary Figure 12 | Potassium atom adsorption on the B-2√3Sn surface imaged at 60 K. STM image of the B-2√3Sn surface before (**a**) and after (**b**) adsorption of ~ 0.005 ML of potassium. Both images are scanned with a dual bias mode (see Supplementary Note 5) with a tunneling bias of ±1.5 V and a tunneling current of 200 pA for (**a**) and 20 pA for (**b**). The scale bars in (**a**) and (**b**) are 10 nm. The smaller current for (**b**) is to reduce the tip-induced hopping of the potassium atoms. 2√3Sn and 4√3Sn areas are labeled in (**a**). Apart from some short streaks due to mobile potassium atoms visible in the image, the adsorbed

potassium atoms mostly produce single bright protrusions in (**b**). These protrusions most likely correspond to single potassium atoms. No clustering of potassium atoms is observed. In (**b**), most areas of the surface exhibit the 2√3Sn phase, while some residual 4√3Sn stripes are marked by dashed rectangles. (**c**) Comparison of $\mathrm{d}I/\mathrm{d}V/(I/V)$ curves of the 2√3Sn phase before and after potassium adsorption, revealing a shift in the chemical potential towards the conduction band after potassium deposition, consistent with electron doping. These data show that electron doping reverses the modulation hole-doping-induced phase transformation.

Supplementary References

[1] Himpsel, F. J., Hollinger, G. & Pollak, R. A. Determination of the Fermi-level pinning position at Si (111) surfaces. *Phys. Rev. B* **28**, 7014-7018 (1983).

[2] González, C., Guo, J., Ortega, J., Flores, F. & Weitering, H. H. Mechanism of the Band Gap Opening across the Order-Disorder Transition of Si(111)(4×1)−In. *Phys. Rev. Lett.* **102**, 115501 (2009).

[3] Nguyen, T. H. *et al.* Coulomb energy determination of a single Si dangling bond. *Phys. Rev. Lett.* **105**, 226404 (2010).

[4] Mönch, W. *Semiconductor surfaces and interfaces* Vol. **26** (Springer Science & Business Media, 2013)

[5] Törnevik, C. *et al.* Adsorption of Sn onSi (111) 7×7: reconstructions in the monolayer regime. *Surf. Sci.* **314**, 179-187 (1994).

[6] Eriksson, P. E. J., Osiecki, J. R., Sakamoto, K. & Uhrberg, R. I. G. Atomic and electronic structures of the ordered 2√3×2√3 and molten 1×1 phase on the Si (111): Sn surface. *Phys. Rev. B* **81**, 235410 (2010).

[7] Ichikawa, T. Structural study of ultrathin Sn layers deposited onto Ge (111) and Si (111) surfaces by RHEED. *Surf. Sci.* **140**, 37-63 (1984).

[8] Sugimoto, Y., Abe, M., Hirayama, S. & Morita, S. Highly resolved non-contact atomic force microscopy images of the Sn/Si(111)-(2√3×2√3) surface. *Nanotechnology* **17**, 4235-4239 (2006).

[9] Srour, W. *et al.* Ultrafast atomic diffusion inducing a reversible (2√3×2√3)R30°↔(3×3)R30° transition on Sn/Si (111):B. *Phys. Rev. Lett.* **114**, 196101 (2015).

[10] Ichikawa, T. & Cho, K. Structural Study of Si (111)(2√3× 2√3) R30°–Sn Surfaces. *Japan. J. Appl. Phys.* **42**, 5239-5245 (2003).

[11] Mayer, A., Salopaasi, H., Pussi, K. & Diehl, R. D. A novel method for the extraction of intensity–energy spectra from low-energy electron diffraction patterns. *Comput. Phys. Commun.* **183**, 1443-1447 (2012).

[12] EasyLEED—Low Energy Electron Diffraction I(E)-spectra Analysis. http://andim.github.io/easyleed/

[13] Ono, M. *et al*. Dimer buckling of the Si (001) 2×1 surface below 10 K observed by low-temperature scanning tunneling microscopy. *Phys. Rev. B* **67**, 201306 (2003).

[14] Polei, S. *et al.* Structural transition in atomic chains driven by transient doping. *Phys. Rev. Lett*. **111**, 156801 (2013).

[15] Zhang, H. *et al*. Stabilization and manipulation of electronically phase-separated ground states in defective indium atom wires on silicon. *Phys. Rev. Lett.* **113**, 196802 (2014).

[16] Ren, X. Y., Kim, H. J., Niu, C. Y., Jia, Y. & Cho, J. H. Origin of Symmetric Dimer Images of Si (001) Observed by Low-Temperature Scanning Tunneling Microscopy. *Sci. Rep.* **6**, 27868 (2016).

[17] Sagisaka, K. & Fujita, D. Emergence of p(2×2) on highly doped n-type Si(100) surfaces: A scanning tunneling microscopy and spectroscopy study. *Phys. Rev. B* **71**, 245319 (2005).

[18] Colonna, S., Ronci, F., Cricenti, A., & Le Lay, G. Metallic Nature of the α− Sn/Ge (111) Surface down to 2.5 K. *Phys. Rev. Lett*. **101**, 186102 (2008)

[19] Spadafora, E. J. *et al.* Identification of surface defects and subsurface dopants in a delta-doped system using simultaneous nc-AFM/STM and DFT. *J. Phys.Chem. C* **118**, 15744-15753 (2014).

[20] International Tables of Crystallography, 2016 Edition; http://it.iucr.org
